# Ka’u Community Asthma Management Program

**DOI:** 10.31372/20200503.1091

**Published:** 2020

**Authors:** Luzviminda Banez Miguel

**Affiliations:** University of Hawai’i Community College, Hilo, Hawai’i, United States

**Keywords:** asthma evidence-based practices, asthma Ka’u program, asthma Hawai‘i program, asthma management, asthma quality improvement, asthma community program, asthma community management program, asthma, asthma training

## Introduction

The “Ka’u Community Asthma Management Program” (KCAMP) is a quality improvement and evidence-based practice Doctor of Nursing Practice (DNP) project. KCAMP’s objective was to determine whether community-based asthma education, self-management, self-efficacy, an asthma action plan, journal writing, and use of peak flow meters reduce asthma exacerbations. The literature supports these effective interventions for asthma control (Andrews, Jones, & Mullan, 2014; Chen, Sheu, Chang, Wang, & Huang, 2010; Federman et al., 2013). KCAMP was designed with community-based interventions to improve the practice of management of asthma, decrease hospital and doctors’ visits’ costs, and improve the lives of people who have asthma. Fourteen adult residents with asthma from the Ka’u District, ages 28 to 75, participated in the program. There were 64% (*n* = 9) females and 36% (*n* = 5) males. The racially diverse group included ten Hawaiians, three Asians, and one Caucasian.

## Problem/Significance of Topic

According to the Centers for Disease Control and Prevention (CDC, 2013), asthma has debilitating effects which impact many people’s mental, physical, and socioeconomic aspects throughout the world. Rural Ka’u district has the highest number of adults who have asthma on the island of Hawai‘i and has limited access to healthcare (Hawai‘i Behavioral Risk Factor Surveillance System, 2014; Health Resources and Services Administration, 2020; State of Hawai‘i Department of Health, 2014). Access to healthcare is also a contributing factor. Although the Ka’u district has a hospital, this facility is mainly for long-term care patients and can only provide immediate emergency care. After receiving initial care in the Ka’u Hospital emergency room, patients needing additional respiratory treatments are transferred to Hilo Medical Center, approximately 60 miles away. This distance often separates families from the asthma patient. These reasons, coupled with the absence of an asthma management program in the district (Hawai‘i State Asthma Control Program, 2014), made it imperative to develop an appropriate community program.

## Methods

Conceptual framework ACE Star Model of Knowledge Transformation (Stevens, 2004) complimented by the community-based participatory approach guided KCAMP. ACE Star Model of Knowledge Transformation’s five stages of discovery of research, evidence summary, translation, integration, and evaluation were used to design, implement, and evaluate KCAMP. Community-based participatory approaches provided opportunities between community members and practitioners through shared leadership, co-teaching, and co-learning (Kulbok, Thatcher, Park, & Meszaros, 2012; Tse, Palakiko, Daniggelis, & Makahi, 2015). Community participation involved the engagement of staff at Hui Mālama Ola Nā ‘Ōiwi Native Hawaiian Health Care Clinic. After a discussion with the clinic staff, an agreement with the objectives of KCAMP was achieved; Hui Mālama Ola Nā ‘Ōiwi staff referred clients to participate in the project. Hui Mālama Nā ‘Ōiwi recruited 14 of their clients with asthma who lived in Ka’u for the program. There were nine (9) females and five (5) males whose ages ranged from 28 to 75 years. The racially diverse group included ten Hawaiians, three Asians, and one Caucasian.

DNP student implemented KCAMP from August to December 2015 with the assistance of Hui Mālama Ola Nā ‘Ōiwi staff and community volunteers. The DNP student provided group and one-on-one home visits, asthma educational teaching, and skills training sessions. The nine (9) participants attending the group had five sessions, and five (5) participants in one-on-one home visits received four (4) sessions. The one-on-one home visits received only four (4) sessions because the orientation session and first asthma education class were combined. The group sessions were held in Na‘ālehu, Hawai‘i at Hui Mālama Ola Nā ‘Ōiwi Native Hawaiian Health Care Clinic and the Hawai‘i County Economic Opportunity Council Center. The participants who attended the KCAMP group sessions arrived at and from the center and clinic with their private transportations or were transported to and from their homes using the Hui Mālama Ola Nā ‘Ōiwi free van services. Asthma education home visits were not a common practice in Ka’u. Unconventional one-on-one home visits were provided due to some participants’ preferences and schedules. Visits were arranged by the DNP student with the participants, either at their homes or at their location choice. This approach allowed them to participate in KCAMP sessions at their convenience. The group sessions lasted two (2) to three (3) hours, depending on how many questions, assistance, and breaks the participants needed. The one-on-one session lasted one (1) to two (2) hours, depending on how many questions and support the clients needed. Components of asthma education included learning about asthma, asthma triggers, and management. Materials for the education sessions included Asthma 101 pamphlets donated by the American Lung Association (ALA) (ALA, 2012). The asthma self-management skills training involved using a peak flow meter and spacers, calculating green, yellow, and red zones, and instructions on keeping an asthma diary. All participants received the same content. Most of the educational content was printed in English, with some materials such as managing asthma printed in Tagalog and Ilocano. Verbal translations of English words to Hawaiian words were done when teaching the participants. Examples of the words used were breath, which is “*hā*” in the Hawaiian language, and to breath, which is “*hanu*” in the Hawaiian language.

## Results

Data were collected from participants’ Asthma Control Test (ACT), Mini Asthma Quality of Life Questionnaire (MiniAQLQ), and asthma educational: knowledge (using a modified ALA Asthma 101) pretest and posttest questionnaire (ALA, 2012); peak flow meter measurements; forced expiratory volume (FEV1) scores; asthma diary; verbal reports about asthma status; and a survey asking participants “which is the most helpful intervention in managing their asthma.” Data were collected, analyzed, and compared from the initial to the final session. Participants’ asthma educational: knowledge posttest questionnaire demonstrated a 93% increase of asthma knowledge from their pretest scores of 64%; ACT posttest scores increased to 100% from 21%, and MiniAQLQ posttest scores increased to 93% from 50%. Final peak flow measurements increased to 86% from 64%, and FEV1 levels increased by 64% from participants’ individual FEV1 levels taken from their first sessions. A survey asking participants, “which is the most helpful intervention in managing their asthma,” yielded 71% choosing asthma education as the most helpful intervention.

## Discussion

The KCAMP objective of reducing exacerbations was met. Participants demonstrated increased asthma knowledge, improved asthma self-management, and supported self-efficacy as the participants gained the confidence to manage their asthma, decreased doctors’ visits, and increased quality of life. Additional evidence that KCAMP participants had increased quality of life was a 14% decrease from 43% in asthma attacks in three months; none of the participants reported having visited their doctors in three months compared to 43% at the beginning of KCAMP. None of the participants reported having to visit the emergency room or were hospitalized because of asthma exacerbations in three months, compared to 14% at the beginning of the project. Oral reports and journal entries of the participants also revealed “*feeling less stress*,” “*feeling better*,” “*life was good in general*,” “*singing better*,” “*hold notes longer*,” “*breathing better*,” taste their food better, made their throats feel better which showed improvements in the quality of life among the participants. Using KCAMP by DNP student and its unique methodology of small group and one-on-one session for key asthma education and asthma self-management skills training were suitable to a diverse population in the Ka’u district of Hawai‘i Island. The project was appropriate in using the collaborative effort of the Ka’u community members. The community members and staff of Hui Mālama Ola Nā ‘Ōiwi provided familiarity, cultural competence, support, connected the participants, and helped the DNP student gain the trust of the participants. The one-on-one teaching session given to participants provided client-centered teaching and learning and allowed flexibility. The DNP student was able to adjust the lessons appropriate to the individual needs and preferences.

Limitations to the project may involve the small sample size and the air quality in Ka’u District during the months of August to December 2015. The Ka’u District is an area that is prone to sulfurous air pollution called, “vog” from the ongoing eruption of K?lauea volcano (Longo et al., 2010) from January 3, 1983, to May 17, 2018 (Bagley, 2018).

## Recommendations

The KCAMP project successfully served as a form of an asthma self-management program tailored to a small, diverse group of clients with asthma in a rural community setting. The program’s components, as well as barriers and challenges of the project, serve as exemplars for nurses and other healthcare professionals to consider in the creation of a similar community program. KCAMP utilization of standardized asthma questionnaires and materials created by the ALA provided reliability and validity. Future consideration is to provide more asthma educational materials geared to the ethnic population. However, additional investigation is needed in determining the reliability and validity of such asthma educational materials before utilization.
